# *I*_h_ Equalizes Membrane Input Resistance in a Heterogeneous Population of Fusiform Neurons in the Dorsal Cochlear Nucleus

**DOI:** 10.3389/fncel.2016.00249

**Published:** 2016-10-27

**Authors:** Cesar C. Ceballos, Shuang Li, Antonio C. Roque, Thanos Tzounopoulos, Ricardo M. Leão

**Affiliations:** ^1^Department of Physiology, Ribeirão Preto Medical School, School of Medicine, University of São PauloRibeirão Preto, Brazil; ^2^Department of Physics, School of Philosophy, Sciences and Letters, University of São PauloRibeirão Preto, Brazil; ^3^Department of Otolaryngology, School of Medicine, University of Pittsburgh, PittsburghPA, USA; ^4^Department of Neurobiology, School of Medicine, University of Pittsburgh, PittsburghPA, USA

**Keywords:** cochlear nucleus, subthreshold conductances, membrane resistance

## Abstract

In a neuronal population, several combinations of its ionic conductances are used to attain a specific firing phenotype. Some neurons present heterogeneity in their firing, generally produced by expression of a specific conductance, but how additional conductances vary along in order to homeostatically regulate membrane excitability is less known. Dorsal cochlear nucleus principal neurons, fusiform neurons, display heterogeneous spontaneous action potential activity and thus represent an appropriate model to study the role of different conductances in establishing firing heterogeneity. Particularly, fusiform neurons are divided into quiet, with no spontaneous firing, or active neurons, presenting spontaneous, regular firing. These modes are determined by the expression levels of an intrinsic membrane conductance, an inwardly rectifying potassium current (*I*_Kir_). In this work, we tested whether other subthreshold conductances vary homeostatically to maintain membrane excitability constant across the two subtypes. We found that *I*_h_ expression covaries specifically with *I*_Kir_ in order to maintain membrane resistance constant. The impact of *I*_h_ on membrane resistance is dependent on the level of *I*_Kir_ expression, being much smaller in quiet neurons with bigger *I*_Kir_, but *I*_h_ variations are not relevant for creating the quiet and active phenotypes. Finally, we demonstrate that the individual proportion of each conductance, and not their absolute conductance, is relevant for determining the neuronal firing mode. We conclude that in fusiform neurons the variations of their different subthreshold conductances are limited to specific conductances in order to create firing heterogeneity and maintain membrane homeostasis.

## Introduction

Theoretical and experimental evidence suggests that the expression levels of different ion channels and conductances vary across individual neurons with similar firing properties (Prinz et al., 2004; Marder and Goaillard, 2006; Goaillard et al., 2009), showing that similar firing properties can be achieved by different combinations of ion channel densities. On the other hand, specific and coordinated correlations of ion channels can be used to maintain constant neuronal behavior (Goldman et al., 2001; Schulz et al., 2006, 2007; Sobie, 2009; Taylor et al., 2009; Grashow et al., 2010; Khurana et al., 2011; Ransdell et al., 2013) suggesting that compensatory mechanisms act to maintain stable physiological properties (O’Leary et al., 2013). Additionally, the role of a specific conductance on the membrane excitability and passive properties depends on the dynamics of the other conductances expressed in the neuron (Ma and Koester, 1996; Swensen and Bean, 2003; Day et al., 2005; Marder et al., 2014). Thus, the modulation of a specific conductance can impact the neuronal membrane properties depending on the environment it is included (Marder et al., 2014). However, if the levels of different conductances in a neuron follow specific rules to maintain stable electrophysiological properties or the neuron can attain the same membrane characteristics using several combinations of densities of different ion channels is still a matter of debate.

To address this question, we studied the principal neuron of the dorsal cochlear nucleus (DCN) (Zhang and Oertel, 1994), the fusiform neuron. Fusiform neurons are divided in two populations based on their firing properties: one presenting spontaneous firing at rest, termed active, and another one which does not produce spontaneous firing, named quiet (Leao et al., 2012; Zugaib et al., 2016). These two firing states are produced by differential expression of an intrinsic ionic conductance (Leao et al., 2012) namely, the differential expression of an inwardly rectifying potassium current (*I*_Kir_), which sets the resting membrane potential (RMP) at different voltage determining whether fusiform neurons fire spontaneously or not. Therefore, in the DCN fusiform neurons intrinsic variations of a specific conductance creates firing heterogeneity, and not diverse variations of different conductances in individual neurons. On the other hand, we demonstrated experimentally and theoretically that variations in the expression of a persistent sodium current (*I*_NaP_), are not relevant for the creation of these two firing modes (Leao et al., 2012). However, we do not know how the other subthreshold conductances present in the DCN fusiform neuron affect the membrane properties of this neuron, more specifically, if variations in their expression correlates with the firing mode and subthreshold membrane properties.

To address this question, we attempted to investigate the role of the two other subthreshold conductances expressed by the DCN fusiform neurons, the hyperpolarization activated cationic current (*I*_h_) and the background leak conductance (*I*_leak_), on the membrane properties and on the creation of the firing modes of these neurons. Additionally using an improved computer model of the fusiform neuron, and quantifying conductance variations on individual neurons, we analyzed the impact of the combination of different sets of conductances on the membrane of the fusiform neurons and how they vary together in order to create the quiet and active phenotypes. We found a specific role of *I*_h_ in equalizing membrane input resistance in these neurons, in response to variations of *I*_Kir_, generating distinct firing modes with similar membrane input resistances.

## Materials and Methods

### Brainstem Slices Preparation and Electrophysiology

ICR or Swiss mice (P17–P25) animals were sacrificed after isoflurane inhalation, according to methods approved by the Institutional Animal Care and Use Committee of the University of Pittsburgh and the Committee on Ethics in Animal Experimentation (CETEA) from the University of São Paulo. Coronal slices containing DCN were obtained as in Zugaib et al. (2016). Briefly brains were removed and cut in cold solution whose composition in mM was: NaCl (87), NaHCO_3_ (25), KCl (2.5), NaH_2_PO_4_ (1.25), CaCl_2_ (0.5), MgCl_2_ (7), glucose (25), sucrose (75), 335 mOsm/kgH_2_O, pH 7.4 when bubbled with carbogenic mixture (95% O_2_ and 5% CO_2_). Before removing the brain from the skull, the vestibulocochlear nerve (VIII cranial nerve) was cut to prevent damage to the DCN. Coronal slices (200 μm thick) containing the three layers of the DCN with its basic circuit (Oertel and Young, 2004) were obtained on a vibratome (Vibratome 1000 Plus) and incubated at 35°C for 45 min and subsequently at room temperature in recording solution [artificial cerebrospinal fluid (aCSF)], whose composition in mM was: NaCl (125), KCl (2.5), NaHCO_3_ (25), NaH_2_PO_4_ (1.25), glucose (10), CaCl_2_ (2), MgCl_2_ (1), 305 mOsm/kgH_2_O, pH 7.4 when bubbled with carbogenic mixture (95% O_2_ and 5% CO_2_). Alternatively, slices were obtained using a Leica 1000S vibratome in warm (35°C) aCSF, stored at this temperature for 1 h and then at room temperature as in Tzounopoulos et al. (2004). No difference was observed between the protocols or mice strain. Single cells were visualized with IR interference contrast optics and recorded using patch pipettes in either voltage- or current-clamp modes. Fusiform neurons were identified based on their electrophysiological and morphological characteristics (for more details see Tzounopoulos et al., 2004; Zugaib et al., 2016). Pipettes were filled with a K^+^-based internal solution containing (in mM): 113 K-gluconate, 4.5 MgCl_2_, 14 tris-phosphocreatine, 9 HEPES, 0.1 EGTA, 4 Na-ATP, 0.3 tris-GTP, 10 sucrose, pH 7.3, ∼300 mOsmol. In some experiments external sodium chloride was replaced by *N*-methyl-D-glucamine chloride (NMDG-Cl).

Whole-cell recordings were performed at 33–36°C using an inline heating system (Warner Instruments) and perfused with aCSF at a rate of approximately 1 ml/min. Data was acquired at 10 or 20 kHz and low-pass filtered at 3 kHz (Bessel) using a MultiClamp 700B connected to a Digidata 1440A board (Axon Instruments) or an EPC-10 amplifier (HEKA Elecktronics). Voltage clamp experiments were performed at a holding potential of -65 mV, while current-clamp experiments were performed at I = 0 in quiet neurons and after injection of negative DC current (-20 to -200 pA) in active neurons. Acceptable access resistance was considered to be below 20 MΩ and was monitored during the whole experiment.

### Computer Simulations

A single compartment model of the fusiform cell was built using a standard Hodgkin–Huxley formalism. It is based on a previous model reported in Leao et al. (2012) containing the following ionic conductances: *I*_Na_ (fast sodium current), *I*_Kd_ (delayed rectifier potassium current), *I*_h_, *I*_NaP_, *I*_Kir_, and an *I*_leak_. The kinetics of time and voltage-dependent parameters were determined by activation and inactivation gating variables as described in Leao et al. (2012). The maximal conductance densities of *I*_Na_ and *I*_Kd_ were adjusted in order to set firing frequencies closer to the experimental observations (Leao et al., 2012), with values of *g*_Na_ = 80 mS/cm^2^ and *g*_Kd_ = 20 mS/cm^2^. The other conductance densities were: *g*_NaP_ = 0.1, *g*_h_ = 0.54, and *g*_leak_ = 0.15 mS/cm^2^. *g*_Kir_ was set at 0.5 mS/cm^2^ for active neurons and at 1 mS/cm^2^ for quiet neurons. The reversal potentials were set at 50 mV for *E*_Na_, -81.5 mV for *E*_K_, -43 mV for *E*_h_, and -51.32 mV for *E*_leak_. The *K*_IR_ time constant used was 0.5 ms.

*I*_h_ was built on a previous description of the current (Nagtegaal and Borst, 2010). We used two activation variables (*A*_h1_ and *A*_h2_) that were described with same steady state dynamics (*A*_h_^∞^) (Leao et al., 2012) but different voltage dependence of time constant (τ_h1_ and τ_h2_). The two activation time constants were used as in Destexhe and Babloyantz (1993). The kinetic equations were as follows

$$I_{h}=g_{h}(0.5*A_{h1}+0.5*A_{h2})*\left(V-E_{h}\right)$$

$$A_{h1}^{\infty }=A_{h2}^{\infty }=\frac{1}{1+e^{(V+87)/8.9}}$$

$$\tau_{h1}=100+e^{(V+183.6)/30.48}$$

$$\tau_{h2}=700\frac{e^{(V+188.6)/11}}{1+e^{(V+105)/5.5}}$$

Based on the experimental data (Leao et al., 2012), the model cell for the quiet versus spontaneously active states differed only in the maximum conductance of *I*_Kir_. The geometry of the model was a cylinder 20 μm of diameter and 20 μm of length. The cell specific capacitance was set at 1 μF/cm^2^. Simulations were run using the NEURON simulator, version7.1, in an 8-core Intel7 processor. The time step was 0.1 ms and the initial membrane potential -65 mV. All measurements were done after waiting 4 s to achieve steady state values. Parameter spaces were obtained varying only two parameters at a time, with the conductance values in the range between 0 and twice their original values. Data was saved and analyzed using MATLAB. In order to determine the regions in parameter space where active and quiet neurons were placed, 1 s of spontaneous activity (i.e., without injected current) was measured. To determine the RMP both sodium conductances (*g*_NaP_ and *g*_Na_) were set to 0 and the RMP was measured at the end of a 1 s sweep over the range of values with no injected current.

The model and simulation files are available for public download on the freely available repository ModelDB^1^.

### Dynamic Clamp

We simulated *I*_h_ using the Real Time Application Interface for Linux-based (RTAI^2^) dynamic clamp (Dagostin et al., 2015). Two computers were used, one for data acquisition running PatchMaster (Heka Elecktronics), and a second ‘dynamic-clamp’ computer that reads voltage from the patch-clamp amplifier (EPC-10, HEKA Elecktronics) and generates current commands in real-time every 50 μs. The ‘dynamic-clamp’ computer is an x86 architecture computer (Pentium 4, Intel) with a PCI-6036E data acquisition card (National Instruments) for reading voltage and generating current commands to the clamped neuron. The real-time dynamic clamp software was written (by Dr. R. N. Leao, Federal University of Rio Grande do Norte, Brazil) in GNU-C, and routines for data acquisition were programmed using the Linux Control and Measurement Device Interface (COMEDI^3^).

### Data Analysis

**FIGURE 1:**
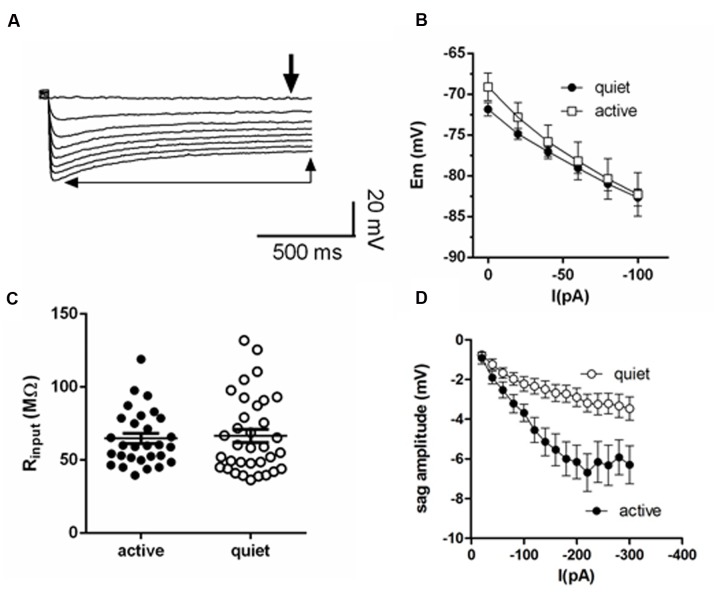
**Membrane input resistance is similar in quiet and active fusiform neurons.** **(A)** Example of the membrane response of a fusiform neuron to hyperpolarizations. The thick arrow shows the point where we measured the membrane potential used to calculate membrane input resistance. The thin arrows show the steady-state level of the membrane potential used to calculate the depolarization sag of the membrane. **(B)** Graph showing the steady-state response of the membrane potential to injected negative DC current in quiet (*n* = 16) and active (*n* = 14) fusiform neurons. **(C)** Summary of the membrane input resistance obtained by the linear regression of the data in **(B)**. **(D)** Graph showing the dependence of the membrane sag amplitude to the current injected. *n* = 15 each.

All data are corrected for a measured liquid junction potential of 10 mV. Active and quiet neurons were classified as in Leao et al. (2012). Fusiform neurons were classified as active when their spontaneous action potential firing rate was >0.5 Hz. Fusiform neurons that did not display any spontaneous firing or that displayed sparse spontaneous firing, with rates below 0.5 Hz, were classified as quiet. RMP was determined in the presence of TTX. The depolarization sag of the membrane was the difference between the steady state and the hyperpolarization peak (**Figure 1A**).

The inward rectifying cationic current (*I*_h_) was elicited by 4 s hyperpolarizations from -65 to -120 mV (in some cells we used -20 mV step hyperpolarizations from -60 to -120 mV) and quantified by subtracting the currents before and after the application of ZD7288 (20 μM). *I*_h_ was measured at the steady state current at the end of the hyperpolarization pulse. Alternatively, *I*_h_ was estimated as the difference between the onset and steady-state current. The conductances obtained using these two approaches were not significantly different (*p* > 0.05). The activation and deactivation of *I*_h_ was measured fitting a double exponential function to the current activation and tail current deactivation. The voltage dependence of *g*_h_ was calculated using the peak amplitude of the tail currents elicited after a repolarization to -65 mV and fitted with a Boltzmann function. Leak currents were defined as the currents left after blocking *I*_h_ and *I*_Kir_ with ZD7288 (20 μM) and Ba^++^ (200 μM), respectively, and *I*_NaP_ with TTX (1 μM) (Leao et al., 2012). The slope conductance was determined using the linear part of the subthreshold IV relationship. Membrane input resistance was calculated both in current-clamp mode as the slope of the VI curves in response to -20 pA steps from 0 to -100 pA (**Figure 1A**), and in voltage-clamp mode as the inverse of the slope of the IV curves from -65 to -80 mV, values close to the RMP of the fusiform neurons (-60 to -80 mV; Leao et al., 2012).

### Drugs

Drugs were prepared from 1000 × stock solutions and diluted before applications. ZD7288 was obtained from Tocris and Ascent Scientific. *N*-methyl D-glucamine-Cl (NMDG), BaCl_2_ and tetrodotoxin (TTX) were purchased from Sigma.

### Statistics

The membrane input resistances were submitted to a normality test (D′Agostino and Pearson) and they were not considered to represent a normal distribution (*p* < 0.0001, *n* = 120) so they were analyzed using a non-parametric test (Mann–Whitney). The other parameters had less data than necessary to apply normality tests (<<100) and were treated as having a normal distribution and their means compared with paired and unpaired *t*-test. However, comparing the data using both parametric and non-parametric tests did not affect our conclusions. Multiple comparisons were performed with one-way ANOVA and a Tukey’s multiple comparison test. Two-tailed significance level was set below 0.05. Correlations were determined using a linear regression fit. Statistics was performed using GraphPad Prism.

## Results

### Active and Quiet Neurons Have Similar Membrane Input Resistances

Quiet fusiform neurons display increased expression of *I*_Kir_, which would produce a smaller membrane input resistance when compared with the input resistance of active neurons. However, we observed that both quiet and active neurons have similar membrane input resistances when measured both in current clamp (quiet: 77.8 ± 8 MΩ; active: 89.1 ± 9 MΩ; *p* = 0.36; *n* = 37 and 36, respectively. **Figures 1B,C**) and in voltage-clamp mode (quiet: 95 ± 12 MΩ; active 77.1 ± 10 MΩ; *p* = 0.09; *n* = 21 and 26, respectively) suggesting the presence of differential expression of compensating conductances that maintain the input resistance constant.

### Fusiform Neurons Express a More Robust *I*_h_ in Active than in Quiet Neurons

Consistent with this hypothesis, we found that active neurons show bigger hyperpolarization sag of the membrane potential (peak sag: -2.9 ± 0.5 mV, quiet; -6.2 ± 0.8 mV, active; *p* < 0.01; *n* = 15 each; **Figure 1D**). Because this sag is inhibited by the hyperpolarization-activated cationic current (*I*_h_) antagonist ZD7288 (not shown), this suggests the presence of a bigger *I*_h_ in these neurons that may normalize the input resistance of quiet and active neurons.

**FIGURE 2:**
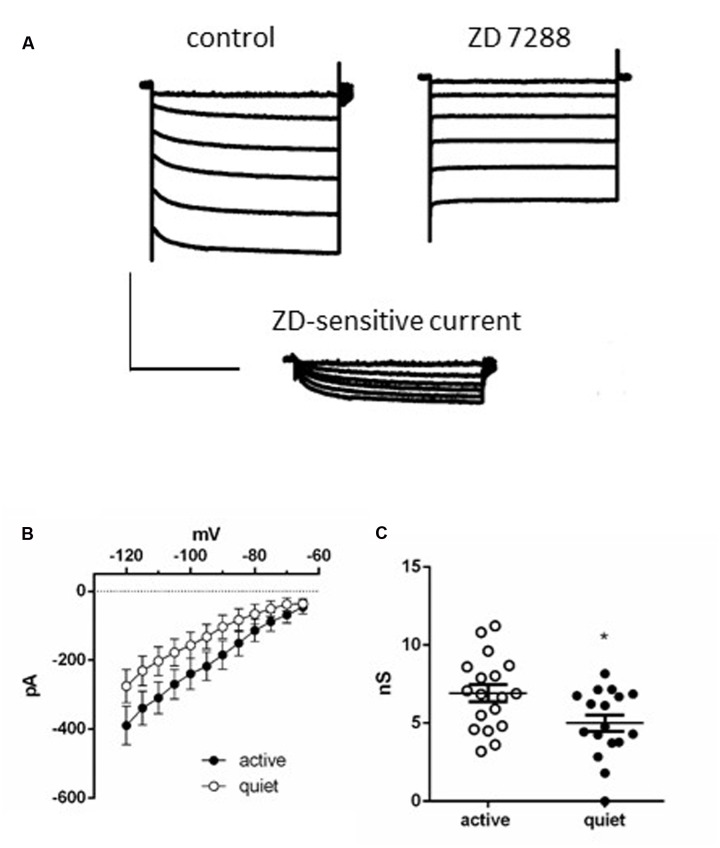
***I*_h_ in DCN fusiform neurons.** **(A)** Example of currents activated by a membrane hyperpolarization before (left) and after (middle) application of ZD7288 (20 μM). Bottom: subtraction of the control currents by the same currents after application of ZD 7288. **(B)** Current-voltage relationships of the ZD 7288 sensitive current (*I*_h_) in quiet and active fusiform neurons. **(C)** Summary of the slope conductances of *I*_h_ in quiet and active fusiform neurons. ^∗^*p* < 0.05.

To further test this hypothesis, we measured *I*_h_ in active and quiet neurons to compare this conductance in both neuronal types. In accordance with the presence of the h current we observed after applying successive hyperpolarizations, a gradual slow developing inward current that reached a steady state in ∼3 s, which was inhibited by ZD7288 (**Figure 2A**). The current-voltage (IV) relationship of the ZD-sensitive current of both quiet and active neurons is shown in **Figure 2B** which shows that the *I*_h_ in quiet and active neurons have similar IV relationships. Using the tail currents we calculated the activation curves of *I*_h_ and they presented similar parameters (quiet neurons had a V_50_ of -86.7 ± 4.6 mV and a slope of 8.2 ± 4 while *I*_h_ from active neurons had a V_50_ of -81.5 ± 5 mV and a slope of 7.1 ± 4, *p* = 0.98). We also calculated the activation and deactivation time constants (fast and slow) of *I*_h_ from quiet and active neurons between -100 and -120 mV. Because we did not observe differences of the time constants in these potentials, we averaged the results from these potentials. Except for the fast component of the activation, none of the other parameters was different between quiet and active neurons (**Table 1**).

**Table 1:** Activation and deactivation time constants of *I*_h_ from quiet and active neurons.

	Activation τ fast	Activation τ slow	Deactivation τ fast	Deactivation τ slow
Quiet	279 ± 29 ms (9)^∗^	1877 ± 333 ms (9)	226 ± 29 ms (9)	1163 ± 154 (9) ms
Active	185 ± 29 ms (8)	1440 ± 265 ms (8)	194 ± 34 ms (6)	1464 ± 354 (6) ms


*^∗^*p* < 0.05, unpaired *t*-test (compared with *g*_Kir_ active).*

While *I*_h_ from quiet and active neurons are similar in its kinetics, active neurons presented a bigger slope conductance of h current (measured between -75 and -100 mV) than quiet neurons (quiet: 5.0 ± 0.5 nS; active: 6.9 ± 0.5 nS; *p* = 0.02, unpaired *t*-test *n* = 17 and 18, respectively; **Figure 2C**). Our data show that on the average active fusiform neurons express a bigger *I*_h_ than quiet neurons.

### A Depolarizing Leak Current Generates a Depolarized RMP after *I*_Kir_, *I*_h_, and *I*_NaP_ Blockage, and Is Expressed Equally in Active and Quiet Neurons

**FIGURE 3:**
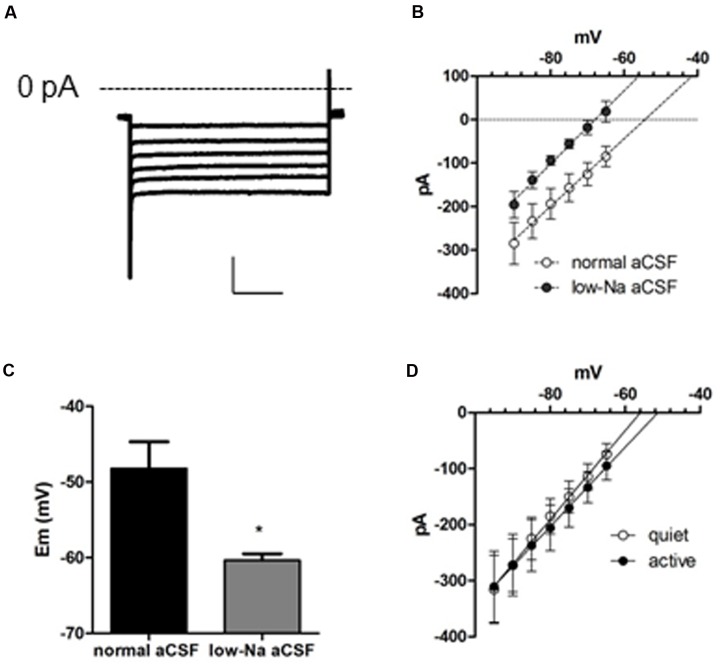
**A background conductance tonically depolarizes quiet and active fusiform neurons.** **(A)** Example of the subthreshold currents evoked by membrane hyperpolarizations after blocking *I*_Kir_, *I*_h_, and *I*_NaP_ with Ba^++^ (200 μM), ZD7288 (20 μM), and TTX (1 μM). **(B)** IV plot of the membrane currents of fusiform neurons recorded in the presence of Ba^++^ (200 μM), ZD7288 (20 μM), and TTX (1 μM) in control aCSF and in low-sodium aCSF. The lines are linear regressions to the data. **(C)** Summary of the effect of a low-sodium aCSF on the resting membrane potential (RMP) of fusiform neurons recorded in the presence of Ba^++^ (200 μM), ZD7288 (20 μM), and TTX (1 μM). ^∗^*p*<0.05, paired *t*-test. **(D)** Plot of the membrane currents of quiet and active fusiform neurons recorded in the presence of Ba^++^ (200 μM), ZD7288 (20 μM), and TTX (1 μM).

To characterize all conductances affecting membrane input resistance, we looked at the background leak currents of the DCN fusiform neuron, which are traditional regulators of membrane passive properties (Enyedi and Czirják, 2010; Renigunta et al., 2015). After inhibition of *I*_Kir_ and *I*_h_ with Ba^++^ (200 μM) and ZD7288, and *I*_NaP_ with TTX (1 μM), a residual current remains in both quiet and active neurons. This current is linear in the range of subthreshold potentials tested, and will be referred as a leak current (**Figure 3A**). Remarkably, this current had a depolarized reversal potential (-49.3 ± 4 mV; *n* = 22). Accordingly, the RMP measured after blockage of *I*_NaP_, *I*_h_, and *I*_Kir_ by TTX, ZD, and Ba^++^, respectively, was similarly depolarized (-49.7 ± 1.7 mV; *n* = 12). These values are considerably above the calculated equilibrium potential of the potassium ions (-84 mV) suggesting that this leak conductance is permeable to cations other than K^+^. Accordingly, perfusion of a low-sodium solution (sodium replaced by NMDG-Cl: [Na^+^]_o_ = 32 mM, *E*_rev_ Na^+^ = 42.6 mV) inhibited the current in all potentials (*p* < 0.001), shifting the reversal potential of the leak current from -49.7 ± 7.3 to -66.2 ± 2.2 mV (**Figure 3B**; *n* = 6) and hyperpolarized RMP from -48.2 ± 3.5 to -60.3 ± 0.9 mV (*n* = 5; *p* = 0.012; **Figure 3C**). We conclude that DCN fusiform neurons express a leak current with Na^+^ permeability that would be capable of depolarizing the membrane potential generating a tonic depolarization if it were not counterbalanced by *K*_ir_ currents.

To determine whether variations on this background conductance could offset the effect on membrane input resistance produced by the smaller *I*_Kir_ in active neurons, we compared this current in quiet and active neurons. We found that both quiet and active neurons presented leak currents of similar magnitudes and reversal potentials (leak conductance: quiet: 8.0 ± 1.7 nS; active: 7.1 ± 1.5 nS; *p* = 0.7; *I*_leak_ reversal: quiet: -52.7 ± 4.9 mV; active: -46.0 ± 6.2 mV; *p* = 0.4; *n* = 13 and 9, respectively; **Figure 3D**). This shows that these background conductances are similar in both fusiform neuronal types and are probably not compensating the difference in membrane input resistance produced by the differential expression of *I*_Kir_.

### *I*_h_ Inhibition Affects RMP Equally in Quiet and Active Neurons but Increases Input Resistance More Prominently in Active Neurons

So far, our results suggest that active neurons express a more robust *I*_h_ in order to compensate for their smaller *I*_Kir_ maintaining a similar input resistance than quiet neurons. If this were true, inhibition of *I*_h_ would reveal a bigger input resistance in active neurons. Also the more depolarized RMP of active neurons (Leao et al., 2012) could be accountable by their bigger *I*_h_. Therefore, we tested the effect of the application of ZD7288 on the RMP and membrane input resistance of quiet and active fusiform neurons.

**FIGURE 4:**
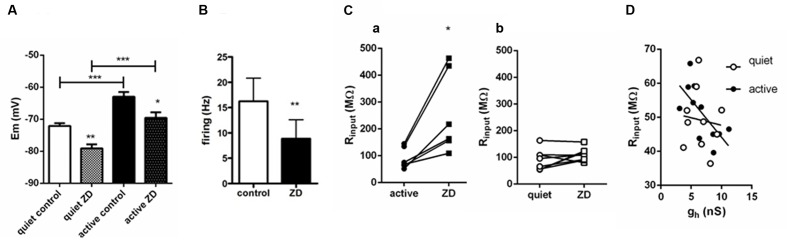
**Influence of *I*_h_ on RMP, firing and input resistance of quiet and active fusiform neurons.** **(A)** Effects of ZD 7288 (20 μM) on the RMP of quiet and active fusiform neurons. ^∗∗^*p* < 0.01; paired *t*-test (compared with active control) and unpaired *t*-test (compared quiet and active ZD); ^∗∗∗^*p*<0.001, paired *t*-test (compared with quiet control). **(B)** Summary of the effect of ZD 7288 on the intrinsic firing of fusiform active neurons. ^∗∗^*p* < 0.01, paired *t*-test. **(C)** Effect of blocking *I*_h_ with ZD7288 on the membrane input resistance measured in voltage-clamp in quiet (a) and active (b) DCN fusiform neurons. ^∗^*p* < 0.05, paired *t*-test. **(D)** Change of membrane input resistance produced by ZD7288 in quiet (*n* = 10) and active neurons (*n* = 7). ^∗∗^*p* < 0.01, unpaired *t*-test. **(E)** Correlation of membrane input resistance with *g*_h_ conductance of quiet and active neurons.

We found that application of ZD7288 affected equally the RMP (measured in the presence of TTX) of active and quiet neurons, hyperpolarizing then by the same amount (quiet: -72.1 ± 0.9 to -79.1 ± 1.3 mV; active: -63 ± 1.5 to -69.6 ± 1.8 mV; differences: quiet: -6.79 ± 1.4 mV; active: -7.6 ± 1.05 mV; *p* = 0.65; *n* = 10 and 7, respectively), maintaining the same difference in RMP in quiet and active neurons observed originally (**Figure 4A**). Experiments performed in the absence of TTX showed that inhibition of *I*_h_ by ZD7288 is efficient in decreasing the spontaneous firing of active neurons (from 16.2 ± 4.5 to 8.9 ± 7 Hz; *n* = 10; *p* < 0.01, paired *t*-test; **Figure 4B**), but unable to convert them to quiet neurons. We conclude that despite the differences in *g*_h_ in active and quiet neurons it does not participate in setting up the RMP differences observed in fusiform quiet and active neurons, and does not define the firing mode of fusiform neurons.

We then measured input resistance of quiet and active neurons before and after application of ZD7288. Because the VI curve of fusiform neurons has a small rectification around -70/-80 mV and the RMP of active and quiet neurons varied at these potentials and is affected by ZD7288, we performed measurements of input resistance in voltage-clamp mode. We found that in contrast to its effect on RMP, ZD7288 had a differential effect on input resistance on quiet and active neurons. While ZD7288 significantly increased R_input_ in active neurons (from 121.6 ± 35 to 314.8 ± 8 MΩ; *p* = 0.007, paired *t*-test, *n* = 7; **Figure 4Ca**), it did not increase significantly membrane input resistance in quiet neurons (from 106.7 ± 22 to 148.5 ± 39 MΩ; *p* = 0.1, paired *t*-test, *n* = 9; **Figure 4Cb**). We conclude that while *I*_h_ has a similar small influence on the RMP of quiet and active neurons, it has, consistent to our prediction, a bigger influence on the membrane input resistance of active neurons.

We previously found that *g*_Kir_ correlates positively with RMP in fusiform neurons, in accordance to its major role in regulating membrane potential. If *g*_h_ is used to compensate for the increased input resistance created by the smaller *g*_Kir_ in active neurons, it is plausible that this conductance would correlate with input resistance in these neurons. In fact, when we correlated *g*_h_ and input resistance in quiet and active neurons we found that *g*_h_ correlated positively only in active neurons (quiet: *r*^2^ = 0.01, *p* = 0.77; active: *r*^2^ = 0.46, *p* = 0.02; **Figure 4D**).

These results show that in active neurons variations in *g*_h_ offsets the difference in the input resistance produced by their smaller expression of *g*_Kir_ and have a membrane input resistance more sensitive to *g*_h_ inhibition than quiet neurons. But despite the difference in *g*_h_ seen in quiet and active neurons its inhibition affected RMP equally in both neurons.

### Active Neurons Are More Sensitive to Artificially Increasing *g*_h_ than Quiet Neurons

**FIGURE 5:**
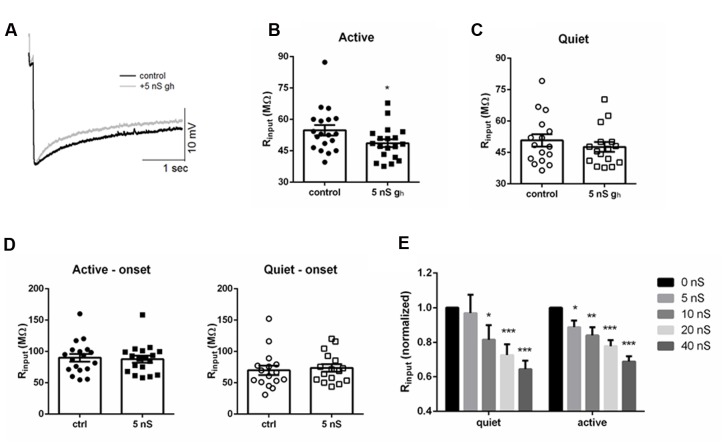
**Injecting artificial *g*_h_ affects differentially membrane input resistance of DCN fusiform quiet and active neurons.** **(A)** Example of the effect of injecting 5 nS of artificial *g*_h_ on the response to hyperpolarizing current in a fusiform neuron. **(B)** Summary of the effect of injecting 5 nS of artificial *g*_h_ on the membrane input resistance of DCN fusiform active neurons. ^∗^*p* < 0.05. **(C)** Summary of the effect of injecting 5 nS of artificial *g*_h_ on the membrane input resistance of DCN fusiform quiet neurons. **(D)** Effect of 5 nS artificial *g*_h_ on the immediate membrane input resistance of fusiform neurons. **(E)** Effect of application of increasing artificial *g*_h_ on the normalized membrane input resistance of quiet and active neurons (*n* = 7 each). ^∗∗^*p* < 0.01; ^∗∗∗^*p* < 0.005.

The bigger sensitivity of the membrane input resistance of active neurons to ZD7288 suggests they are more sensitive to changes in *I*_h_ than quiet neurons. If this were true, the membrane input resistance of active neurons would be more sensitive to increases in *I*_h_ than in quiet neurons. To test this hypothesis more directly we roughly doubled *g*_h_ by injected an artificial *g*_h_ (5 nS) in both quiet and active neurons (**Figure 5A**) and measured their membrane input resistance before and after artificially increasing *g*_h_ in both neuronal types. We found a significant decrease in membrane input resistance in active neurons (**Figure 5B**; *p* = 0.0004, Wilcoxon matched-pairs signed rank test; *n* = 7) while it did not affect significantly the membrane input resistance of quiet neurons (**Figure 5C**; *p* = 0.16, Wilcoxon matched-pairs signed rank test; *n* = 7). No change in input resistance was observed when we measured the input resistance at the onset of the hyperpolarization pulses (**Figure 5D**) accordingly to what is expected for an effect produced by *I*_h_.

We then tested how further injection of subsequent bigger artificial *g*_h_ could affect membrane input resistance of quiet and active neurons. Curiously, doubling *g*_h_ to 10 nS was effective in decreasing input resistance in quiet neurons (**Figure 5E**). When we normalized the input resistance and examined the effect of increasing *g*_h_, we found that the drop in input resistance produced by increasing *g*_h_ was similar in quiet and active neurons above 10 nS (**Figure 5E**), reaching around 70% of the original value at 40 nS of *g*_h_. Interestingly, injection 40 nS of artificial *g*_h_ in quiet neurons produced a small depolarization of the membrane potential (from -58.7 ± 2 to -54.2 ± 1 mV; *p* < 0.05, paired *t*-test; *n* = 7), but did not produce spontaneous firing, showing that *I*_h_ is not effective in depolarizing the membrane to values sufficient to produce spontaneous firing in fusiform neurons.

We conclude that the membrane input resistance of active neurons is very sensitive to adding 5 nS of *g*_h_, roughly doubling *g*_h_, while this did not affect significantly the membrane input resistance of quiet neurons. On the other hand, membrane input resistance of quiet and active neurons is equally sensitive to further applications of increasing artificial *g*_h_.

### A Computer Model Shows that RMP is Strongly Influenced by *I*_Kir_ and *I*_Leak_, and Input Resistance by *I*_h_

**FIGURE 6:**
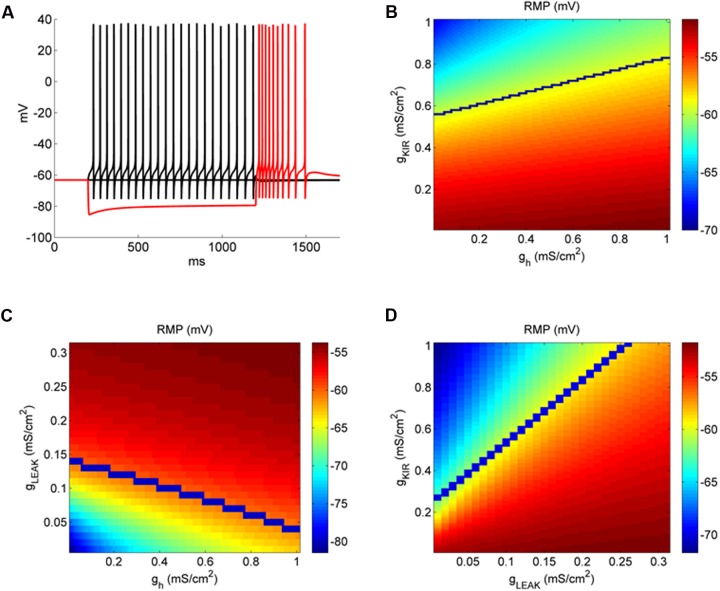
***I*_Kir_ affects more RMP than *I*_h_ in a model of DCN fusiform neuron.** **(A)** Example of the behavior of the fusiform neuron model to membrane hyperpolarization and depolarization. **(B)** Conductance space of *g*_h_ versus *g*_Kir_ affecting RMP (color coded). The blue line represents the transition from quiet (above) to active (below) firing modes. **(C)** Conductance space of *g*_Leak_ versus *g*_h_ affecting RMP (color coded). The blue line represents the transition from quiet (above) to active (below) firing modes. **(D)** Conductance space of *g*_Kir_ versus *g*_Leak_ affecting RMP (color coded). The blue line represents the transition from quiet (above) to active (below) firing modes.

We found that the difference of *g*_h_ of quiet and active neurons is not very big and its inhibition affected their RMP similarly, but membrane input resistance after ZD7288 is considerably bigger in active neurons. This is in accordance with a bigger sensitivity of membrane input resistance of active neurons to variations in *g*_h_. Accordingly, *g*_h_ only correlated with input resistance in active neurons, suggesting that variations in this conductance are used by active neurons to compensate for their smaller *g*_Kir_ and maintain homeostatic control of membrane input resistance. Additionally, this difference in *g*_h_ seems to not to affect the RMP on quiet and active neurons differentially. It seems that the effects of variations of *g*_h_ on membrane properties depend on the level of other conductances differentially expressed in quiet and active neurons. To understand how concerted variations in these currents affect subthreshold membrane properties and the transition quiet to active and membrane input resistance, we used a computer model containing the identified subthreshold conductances (*g*_Kir_, *g*_h_, *g*_leak_) of the fusiform cell (**Figure 6A**).

We found previously (Leao et al., 2012) that RMP of fusiform neurons is very sensitive to *I*_Kir_ inhibition by Ba^++^ 200 μM, and that the differences in its magnitude create the differences of RMP which creates the firing modes of the fusiform neuron. On the other hand, even with a bigger *I*_h_ found in active neurons, its inhibition was not sufficient to change differently the RMP of active and quiet neurons. We then compared how changing *g*_h_ and *g*_Kir_ in our model influenced RMP. In **Figure 6B** we see the effect on RMP of the changing *g*_Kir_ and *g*_h_. It is clear that RMP is much more sensitive to changing in *g*_Kir_ than in *g*_h_. For instance, for a given *g*_h_ (0.5 mS cm^-2^), RMP changes from -52.1 to -63.4 mV varying *g*_Kir_ from 0 to 1 mS cm^-2^, while varying *g*_h_ from the same amount only changed RMP from -58.5 to -55.6 mV from a *g*_Kir_ of 0.5 mS cm^-2^. We also can see that accordingly to its effect on RMP, *g*_Kir_ affects strongly the transition to quiet to active (blue line) in contrast to *g*_h_. Interestingly, it can be seen that the impact of *g*_h_ on RMP is bigger in quiet than active neurons (compare the area above and below the blue line), which is different from what we observed experimentally by inhibiting *I*_h_ with ZD7288 (**Figure 4A**). In **Figure 6C** we analyzed the variations of *g*_leak_ and *g*_h_ (keeping *g*_Kir_ at 0.5 mS cm^-2^). Again, variations of *g*_h_ are less effective in affecting RMP when compared with variations of *g*_leak_, especially in active neurons (region above the blue line, with bigger values of *g*_leak_). Finally we compared the impact of varying *g*_leak_ and *g*_Kir_ on the RMP and quiet-active transition (**Figure 6D**). We see that while increasing *g*_Kir_ can hyperpolarize the RMP, *g*_lleak_ is very effective in depolarize RMP, because we needed around 4–5 times more *g*_Kir_ to counteract the effect of *g*_leak_, and produce a quiet neuron (above the blue line).

**FIGURE 7:**
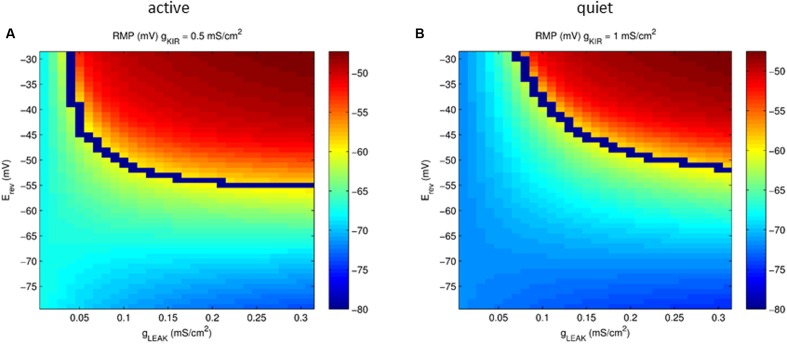
**Plot of the parameter space of the leak conductance versus reversal potential affecting RMP (color coded) in a model neuron with *g*_Kir_ conductances typical of an active **(A)** and quiet **(B)** neuron.** The blue line represents the transition from quiet (below) to active (above) firing modes.

Our model showed that the depolarizing *g*_leak_ has a strong depolarizing effect on RMP. To evaluate the interaction of the reversal potential of the leak current and its conductance in RMP we varied both conductance and reversal potential of *g*_leak_ in model neurons with values of *g*_Kir_ typical of an active and a quiet neuron. **Figure 7** shows that only with a reversal potential around -50 mV we were able to reach the values of activity threshold set by the *I*_NaP_ (-59/-57 mV). Also at values of *E*_rev_ below -65 mV the RMP becomes almost insensitive to variations of *g*_leak_, especially in quiet neurons. Although a bigger *I*_Kir_, as seen in quiet neurons, reduces the impact of *g*_lleak_ on depolarizing RMP, it does not prevent its effect on creating active firing in high conductances of *g*_leak_ (**Figure 7B**). We concluded that the presence of a sodium component that increases the reversal potential of *g*_leak_ is fundamental to depolarize the membrane allowing spontaneous firing in DCN fusiform neurons.

**FIGURE 8:**
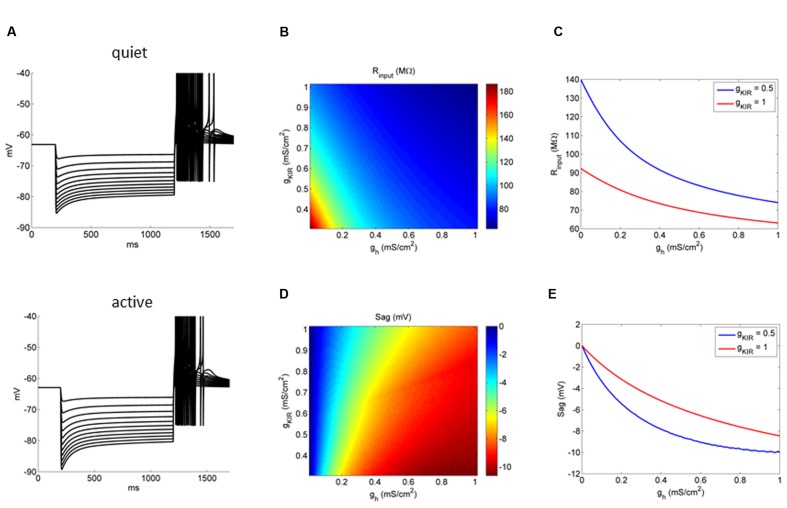
**Influence of *g*_Kir_ on the impact of *g*_h_ on the membrane input resistance and response to hyperpolarizations.** **(A)** Example of the behavior of a quiet and active fusiform neuron model to successive hyperpolarizations. **(B)** Conductance space of *g*_h_ versus *g*_Kir_ affecting membrane input resistance (color coded). **(C)** Two examples showing the effect of increasing *g*_h_ on the membrane input resistance in two models: one with a *g*_Kir_ typical of quiet neurons (red) and the other with a *g*_Kir_ typical of active neurons (blue). **(D)** Conductance space of *g*_h_ versus *g*_Kir_ affecting membrane depolarization sag (color coded). **(E)** Two examples showing the effect of increasing *g*_h_ on the membrane depolarization sag in two models: one with a *g*_Kir_ typical of quiet neurons (red) and the other with a *g*_Kir_ typical of active neurons (blue).

We then studied the relationship of variations of *g*_h_ and *g*_Kir_ on the membrane input resistance of fusiform neurons (**Figure 8A**). The model showed that, similarly to what we observed experimentally, the bigger *g*_Kir_, the smaller the effect of *g*_h_ on membrane input resistance (**Figure 8B**). **Figure 8C** shows the relationship of membrane input resistance and *g*_h_ in two situations: with a low *g*_Kir_ typical of active neurons, and a big *g*_Kir_ typical of quiet neurons. In accordance to what was observed experimentally, *g*_h_ has a more pronounced impact in the membrane input resistance in the model active neuron than in the model quiet neuron. We also observed a smaller depolarizing sag of the membrane potential after hyperpolarization in quiet neurons. This might be caused by a reduced *g*_h_ in these neurons, but because the differences observed in *g*_h_ between quiet and active neurons were not very pronounced, we decided to test if the bigger *g*_Kir_ of quiet neurons dampens the depolarization sag in these neurons. In fact, our model shows that the influence of *g*_h_ in producing the sag is diminished when *g*_Kir_ is bigger (**Figures 8C–E**). Our model and experimental data show that the *g*_h_ and *g*_Kir_ interaction not only controls the membrane input resistance, but also affects dynamic responses of the membrane during hyperpolarization.

### In the Same Cell, *g*_h_ Increases Proportionally as *g*_Kir_ Decreases, Keeping Membrane Resistance Constant

Our model predicts that in a neuron with more prominent *g*_Kir_, *g*_h_ has less impact on membrane input resistance. Accordingly, we found that the membrane input resistance of quiet neurons, which had been shown to express a bigger *g*_Kir_, is less sensitive to inhibition and enhancement of *g*_h_. However, we do not know how exactly both conductances interact in a single cell to produce their effects in the RMP and input resistance. In order to evaluate how these individual conductances, plus *g*_leak_, behave in a single neuron, we compared the subthreshold conductances *g*_Kir_, *g*_h_, and *g*_leak_ in the same neurons (six quiet and six active), by applying in sequence ZD7288 and Ba^++^ in order to compare the proportions of these conductances in the same neuron.

**FIGURE 9:**
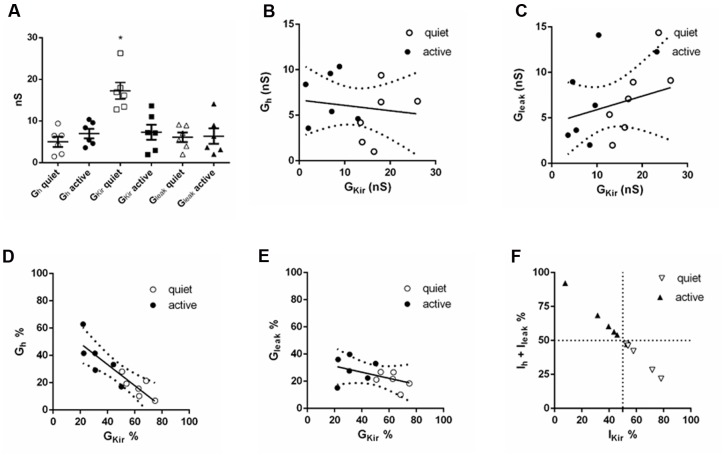
**Comparison of subthreshold conductances in the same neuron.** **(A)** Individual values of *g*_h_, *g*_Kir_ and *g*_leak_ in six quiet and six active neurons. ^∗^*p* < 0.05, unpaired *t*-test (compared with *g*_Kir_ active). **(B)** Correlation of *g*_h_ with *g*_Kir;_ *r*^2^ = 0.02, *p* = 0.66. **(C)** Correlation of *g*_leak_ with *g*_Kir;_ *r*^2^ = 0.07, *p* = 0.39. **(D)** Correlation of *g*_h%_ with *g*_Kir%_; *r*^2^ = 0.77, *p* = 0.0002. **(E)** Correlation of *g*_leak%_ with *g*_Kir%_; *r*^2^ = 0.22, *p* = 0.12. **(F)** Distribution of quiet and active neurons regarding to the proportion of *g*_h +_ *g*_leak_ with *g*_Kir_.

We found, as previously, that *g*_Kir_ was significantly different in quiet and active neurons (**Figure 9A**). *g*_h_ was bigger in active neurons, but the value did not achieve significance (**Figure 9A**). Surprisingly, the absolute values of *g*_Kir_ and *g*_h_ (or *g*_leak_) did not correlate inversely as would be expected if *g*_h_ (or *g*_leak_) increased as *g*_Kir_ decreased in order to keep input resistance similar in the two types (**Figures 9B,C**). However, we found that the proportion of the total subthreshold conductances (*g*_Kir_ + *g*_h_ + *g*_leak_) of *g*_Kir_ and *g*_h_ varied inversely significantly both in quiet and active neurons while *g*_Kir_ and *g*_leak_ did not (**Figures 9D,E**). Accordingly, when we analyzed the proportion of the subthreshold hyperpolarizing conductance (*g*_Kir_) and the depolarizing conductances (*g*_h_ + *g*_leak_), we found that when *g*_Kir_ is responsible for 50% or more of the total subthreshold conductances, the neurons is quiet, while when *g*_Kir_ represents 50% or less of these conductances the neuron is active (**Figure 9F**). We conclude that while the absolute individual values of *g*_h_ and *g*_Kir_ are not inversely correlated in an individual fusiform neuron, their individual proportions vary inversely in order to keep membrane input resistance constant in quiet and active neurons.

## Discussion

Our previous study (Leao et al., 2012) demonstrated that the expression of a large potassium inwardly rectifying current (*I*_Kir_) is necessary to set the membrane potential below the activity threshold, in order to avoid spontaneous firing at rest in quiet neurons. In active neurons, a diminished *I*_Kir_ allowed the RMP to cross the activity threshold producing the spontaneous firing at rest characteristic of active neurons. Therefore, *I*_Kir_ determines the state of the fusiform neuron: quiet or active. Here, we showed that coordinated variations in *I*_h_ keep membrane input resistance constant in quiet and active neurons. We also identified a background depolarizing leak conductance, which consistently depolarizes the membrane of fusiform neurons, producing the depolarization driving of the spontaneous firing of fusiform neurons. However, variations in these two conductances are not important for creating the quiet and active types. Our data show that in DCN fusiform neurons, variations in specific subthreshold conductances contribute differentially to specific features of its excitability.

*I*_h_ is a subthreshold cationic current, produced by HCN channels, which depolarizes the membrane potential when the membrane is hyperpolarized. *I*_h_ can control RMP (Doan and Kunze, 1999; Bal and Oertel, 2000) and rate of firing in several neuronal types (McCormick and Huguenard, 1992; Maccaferri and McBain, 1996; Rodrigues and Oertel, 2006). DCN fusiform neurons of the rat express HCN2 subunit but not HCN1 (Koch et al., 2004), which is consistent with the slow activation of *I*_h_ we found in fusiform neurons. We found that *I*_h_ is not necessary for both spontaneous firing and the depolarized RMP after blocking *I*_Kir_, the latter caused by the presence of a background Na^+^ current. Even though *I*_h_ can modulate the firing of active neurons and the RMP of active and quiet neurons, it could not define a firing type (quiet or active) since *I*_h_ inhibition could not transform an active to a quiet neuron and injection up to 40 nS of an artificial *I*_h_ did not transform a quiet neuron to a firing one. Our computer model demonstrated that *I*_h_ is more appropriate for offsetting membrane input resistance than *I*_leak_, since it affects less RMP, so variations in this conductance will have less impact on the active/quiet transition, than variations in *I*_leak_, which affect more prominently RMP. Thus, variations in *I*_h_ are more appropriate to offset the differences in input resistance caused by variations of *I*_Kir_ in quiet and active neurons, because it does not affect their firing mode.

We found that a modest bigger *I*_h_ in active neurons does not affect substantially RMP but can diminish input resistance enough to compensate for the smaller *I*_Kir_ in active neurons. This can be understood if we take into account the activation of *I*_h_ by hyperpolarization and its reversal potential above RMP. First, the activation of *I*_h_ results of a depolarization of the membrane, but because *I*_h_ deactivates before the current reaches the reversal potential this creates a negative feedback shunting the effect of *I*_h_ in depolarizing the membrane. Additionally since active neurons have a more depolarized RMP than quiet neurons, a smaller percentage of *I*_h_ is activated in active neurons than in quiet ones, which offsets the difference in *I*_h_ magnitude between the neuronal types. On the other hand, because input resistance was measured by hyperpolarizations this effect did not affect this parameter.

On the other hand, our model predicted that *I*_h_ would have a bigger impact on the RMP of quiet neurons, what we did not observe experimentally with ZD7288. Because RMP is much more affected by *I*_Kir_ and *I*_leak_ than *I*_h_, the variations on the RMP produced by variations of these conductances can mask the differential effect of inhibiting *I*_h_ on RMP of quiet and active neurons. Additionally we found in our model that differences in the V_1/2_ of *I*_h_ affects the impact of *I*_h_ on the RMP (not shown), and variations on this parameter are possibly another source of variability. We conclude that although our model still does not explain completely the influence of *I*_h_ on RMP in quiet and active neurons, it is in accordance to most of our experimental observations about the impact of *I*_h_, *I*_Kir_, and *I*_leak_ on the subthreshold properties of the DCN fusiform neurons.

In several neuronal types it has been observed that different conductances can vary differentially in order to attain a homeostatic balance of neuronal excitability (Marder and Prinz, 2002; O’Leary et al., 2013). More specifically a certain neuronal population can maintain a stable firing pattern and specific passive membrane properties across its individual neurons expressing different magnitudes of opposing conductances. For instance in crab stomatogastric neurons concerted variation of *I*_h_ and *I*_KA_ produces changes in gain control maintaining a stable firing pattern (MacLean et al., 2003; Burdakov, 2005). In cerebellar Purkinje neurons there are diverse levels of expression of different conductances producing a similar firing output (Swensen and Bean, 2005). In the auditory system it has been observed that stable firing pattern and RMP can be obtained in neurons from the ventral cochlear nucleus (VCN) by coordinated expression of the opposing h current and a low-threshold potassium current (Cao and Oertel, 2011). Similarly, in dopaminergic midbrain neurons *I*_h_ and A-type potassium currents presented a coordinated variation of their conductances in order to stabilize rebound firing (Amendola et al., 2012). On the other hand, we observed that in DCN principal neurons variations in *I*_h_ are used not to create differences in membrane potential or produce active firing, but to stabilize membrane resistance, a likely homeostatic compensation for the differences in the *I*_Kir_ magnitude in quiet and active neurons (Leao et al., 2012). This was clearly visible when we compared the percentage of each conductance in individual neurons. We found that the percentage of *g*_h_ expressed in an individual neuron varied inversely with the percentage of *g*_Kir_, while *g*_leak_ did not correlate well with both conductances. Thus, in DCN fusiform neurons we have a concerted variation of specific conductances, one to establish different neuronal behaviors (*I*_Kir_) while other (*I*_h_) to keep membrane resistance stable while varying RMP across the fusiform neuronal population. This compensation is important for keeping similar responses to synaptic inputs in both active and quiet neurons, and specially for maintaining similar integration time windows of EPSPs and IPSPs, which are fundamental for expressing long-term plasticity in these neurons (Doiron et al., 2011). Consistent with this, in hippocampal organotypical cultures, chronic inhibition of inhibitory or excitatory neurotransmission homeostatically increases and decreases *I*_h_ expression, respectively, affecting membrane input resistance and EPSP temporal summation and stabilizing long-term potentiation induction in these conditions (Gasselin et al., 2015). Therefore, we propose that the increased *I*_h_ in active neurons is a homeostatic adaptation aimed at equalizing the membrane resistance of both types of fusiform neurons in order to keep membrane responses to synaptic currents similar in both types. Interestingly, down-regulation of *I*_h_ and consequent increased membrane input resistance and more hyperpolarized membrane potential, has been implicated to resilience to tinnitus in noise-exposed mice, while mice which fusiform neurons with decreased KCNQ channel current, but normal *I*_h_, developed tinnitus (Li et al., 2015). This suggests that alterations in the homeostatic control of membrane parameters can affect the response of fusiform neurons to intense sound stimulation and be decisive for the development of tinnitus.

Both our computer model and our experimental data showed that the effect of *g*_h_ in affecting membrane input resistance and the depolarization sag are dependent on the magnitude of *g*_Kir_, more specifically in quiet neurons with a bigger *g*_Kir_, the impact of *g*_h_ on these parameters is smaller than in active neurons. Our data shows that the impact of a specific conductance on the membrane properties is strongly dependent on the membrane “environment” of other conductances. Although this is not a new concept, for instance is well-known how *I*_h_ affects the membrane response to synaptic currents (Magee, 1998; Masi et al., 2015), it is many times overlooked in studies analyzing the impact of a specific conductance on membrane properties. Also, our data shows that the magnitude of the depolarization sag cannot be a reliably parameter to quantify *I*_h_ without knowing the other subthreshold conductances. Similarly, we showed that more important than analyzing the absolute value of the conductance is to measure it in conjunction with other conductances. This was clear when we compared proportions of the conductances in a single neuron, instead of absolute values, that there was a threshold of 50% of the hyperpolarizing conductance (*g*_Kir_) to change the phenotype of the neuron from quiet to active.

Finally, both our data and our model established that the main depolarization drive of DCN fusiform neurons is a linear background current, which presents a fraction permeable to Na^+^. Replacing of most external Na^+^ hyperpolarized drastically the membrane (after *I*_Kir_ blockage) and shifted the reversal potential of the background current accordingly. Sodium permeable leak currents have been identified in several types of neurons (Raman et al., 2000; Lu et al., 2007; Khaliq and Bean, 2010; Lazarenko et al., 2010; Lu and Feng, 2011) driving pacemaker activity by keeping the RMP constantly depolarized. Interestingly neurons from the deep cerebellar nucleus, which would be the cerebellar equivalent of DCN fusiform neurons (Oertel and Young, 2004), also present a Na^+^-permeable background current that keeps the membrane depolarized sustaining spontaneous firing (Raman et al., 2000). A channel that has the properties of a Na^+^ background current has been identified (NALCN; Lu et al., 2007; Ren, 2011), and is a likely candidate for the sodium component of the leak conductance in DCN fusiform neurons. But, because the Na-leak current strongly affects RMP, it could not change with *I*_Kir_ to equalize membrane input resistance without changing the firing mode of the fusiform neuron, which is in accordance with the observation that variations of this conductance do not correlate with the quiet and active modes of firing. However, our model showed that variations in both conductance and reversal potential of this current can produce quiet and active neurons in values of *g*_Kir_ typical of these states. This shows that, physiologically, the parameter space of the variations of *g*_leak_ (and its reversal potential) are probably limited to values which do not affect the firing mode of the DCN fusiform neuron.

We conclude that the DCN fusiform neuron vary their intrinsic subthreshold conductances accordingly to their roles in creating the firing modes, maintaining membrane resistance constant and depolarizing the membrane potential. Our findings show that the “instructions” for creating quiet and active fusiform neurons, follow specific rules, rather than using selected random variations of these conductances resulting in the final “desired” phenotype.

## Author Contributions

RL, TT, and CC designed experiments; RL, CC, and SL performed experiments; CC and AR developed the computational model; RL, CC, and SL analyzed data; RL, CC, AR, and TT wrote the manuscript. All authors approved the final version.

## Conflict of Interest Statement

The authors declare that the research was conducted in the absence of any commercial or financial relationships that could be construed as a potential conflict of interest.

## Footnotes

1 https://senselab.med.yale.edu/ModelDB/showmodel.cshtml?model=206252

2 http://www.rtai.org

3 http://www.comedi.org
