# Corrigendum: *I*_h_ Equalizes Membrane Input Resistance in a Heterogeneous Population of Fusiform Neurons in the Dorsal Cochlear Nucleus

**DOI:** 10.3389/fncel.2017.00010

**Published:** 2017-01-25

**Authors:** Cesar C. Ceballos, Shuang Li, Antonio C. Roque, Thanos Tzounopoulos, Ricardo M. Leão

**Affiliations:** ^1^Department of Physiology, Ribeirão Preto Medical School, School of Medicine, University of São PauloRibeirão Preto, Brazil; ^2^Department of Physics, School of Philosophy, Sciences and Letters, University of São PauloRibeirão Preto, Brazil; ^3^Department of Otolaryngology, School of Medicine, University of PittsburghPittsburgh, PA, USA; ^4^Department of Neurobiology, School of Medicine, University of PittsburghPittsburgh, PA, USA

**Keywords:** cochlear nucleus, subthreshold conductances, membrane resistance, *I*_h_, fusiform neuron

1. In the original article, there was a mistake in 4th Equation on page 3. There should be a plus (“+”) sign after the number 700. So it is an addition, not a multiplication. The authors apologize for the mistake. This error does not change the scientific conclusions of the article in any way.

2. In the original article, the legends of the calibration axis on Figure 2A were missing. They are 500 pA (y-axis) and 4 s (x-axis). The authors apologize for the mistake. This error does not change the scientific conclusions of the article in any way.

3. In the original article, the legends of the calibration axis on Figure 3A were missing. They are 200 pA (y-axis) and 1 s (x-axis). The authors apologize for the mistake. This error does not change the scientific conclusions of the article in any way.

4. In the original article, the legend of panel 4D is wrong. It belongs to a panel that was present in a previous version of the manuscript, and was subsequently deleted. It should be replaced by the legend of panel 4E (which does not exist). This error does not change the scientific conclusions of the article in any way. The correct version of the legend of Figure 4 is:

**Figure 4. Influence of *I*_h_ on resting membrane potential, firing and input resistance of quiet and active fusiform neurons**. **(A)** Effects of ZD 7288 (20 μM) on the resting membrane potential of quiet and active fusiform neurons. ^**^p < 0.01; paired *t*-test (compared with active control) and unpaired *t*-test (compared quiet and active ZD); ^***^p < 0.001, paired *t*-test (compared with quiet control). **(B)** Summary of the effect of ZD 7288 on the intrinsic firing of fusiform active neurons. ^**^p < 0.01, paired *t*-test. **(C)** Effect of blocking *I*_h_ with ZD7288 on the membrane input resistance measured in voltage-clamp in quiet (left) and active (right) DCN fusiform neurons. ^*^p < 0.05, paired *t*-test. **(D)** Correlation of membrane input resistance with g_h_ conductance of quiet and active neurons.

5. In the Figure 9F the axis represent conductances (g) and not currents (I). The authors apologize for the mistake. This error does not change the scientific conclusions of the article in any way.

## Conflict of interest statement

The authors declare that the research was conducted in the absence of any commercial or financial relationships that could be construed as a potential conflict of interest.

